# Making a complex dental care tailored to the person: population health in focus of predictive, preventive and personalised (3P) medical approach

**DOI:** 10.1007/s13167-021-00240-7

**Published:** 2021-04-19

**Authors:** V. V. Tachalov, L. Y. Orekhova, T. V. Kudryavtseva, E. S. Loboda, M. G. Pachkoriia, I. V. Berezkina, O. Golubnitschaja

**Affiliations:** 1 Therapeutic Dentistry and Periodontology Department, Pavlov First Saint Petersburg State Medical University, 6/8 Lva Tolstogo Street, St. Petersburg, Russia; 2 City Periodontology Centre, “PAKS”, Dobrolubova prospect, 27, St. Petersburg, Russia; 3 Predictive, Preventive, Personalised (3P) Medicine, Department of Radiation Oncology, University Hospital Bonn, Rheinische Friedrich-Wilhelms-Universität Bonn, 53127 Bonn, Germany

**Keywords:** Oral cavity, Hygiene, Healthcare, Health policy, Dental diseases, Periodontitis, Collateral pathologies, Patient stratification, Age, Elderly, Motivation, Compliance, Treatment algorithm, Tailored care, Psychological aspects, Dry mouth syndrome, Microbiome, Individualised patient profiling, Socio-economic status, Predictive preventive personalised medicine (PPPM / 3PM), Viral infection, COVID-19, SARS-CoV-2, Pathomechanism, Aggravation, Periodontopathic microflora, Microbiome, Gut-lung axis, Lower respiratory tract, Influenza, Bacterial load, Disease severity, Morbidity, Bacterial superinfections, Inflammation, Comorbidities, Risk factors, Probiotics, Big data, Machine learning, Bio-banking

## Abstract

An evident underestimation of the targeted prevention of dental diseases is strongly supported by alarming epidemiologic statistics globally. For example, epidemiologists demonstrated 100% prevalence of dental caries in the Russian population followed by clinical manifestation of periodontal diseases. Inadequately provided oral health services in populations are caused by multi-factorial deficits including but not limited to low socio-economic status of affected individuals, lack of insurance in sub-populations, insufficient density of dedicated medical units. Another important aspect is the “participatory” medicine based on the active participation of population in maintaining oral health: healthcare will remain insufficient as long as the patient is not motivated and does not feel responsible for their oral health. To this end, nearly half of chronically diseased people do not comply with adequate medical services suffering from severely progressing pathologies. Noteworthy, the prominent risk factors and comorbidities linked to the severe disease course and poor outcomes in COVID-19-infected individuals, such as elderly, diabetes mellitus, hypertension and cardiovascular disease, are frequently associated with significantly altered oral microbiome profiles, systemic inflammatory processes and poor oral health. Suggested pathomechanisms consider potential preferences in the interaction between the viral particles and the host microbiota including oral cavity, the respiratory and gastrointestinal tracts. Since an aspiration of periodontopathic bacteria induces the expression of angiotensin-converting enzyme 2, the receptor for SARS-CoV-2, and production of inflammatory cytokines in the lower respiratory tract, poor oral hygiene and periodontal disease have been proposed as leading to COVID-19 aggravation. Consequently, the issue-dedicated expert recommendations are focused on the optimal oral hygiene as being crucial for improved individual outcomes and reduced morbidity under the COVID-19 pandemic condition. Current study demonstrated that age, gender, socio-economic status, quality of environment and life-style, oral hygiene quality, regularity of dental services requested, level of motivation and responsibility for own health status and corresponding behavioural patterns are the key parameters for the patient stratification considering person-tailored approach in a complex dental care in the population. Consequently, innovative screening programmes and adapted treatment schemes are crucial for the complex person-tailored dental care to improve individual outcomes and healthcare provided to the population.

## Introduction

### Oral cavity health is pivotal for prediction and prevention of associated pathologies

Oral cavity health is in focus of advanced 3PM strategies due to its key role in predicting and preventing dental diseases which may further cascade associated systemic effects and pathologies [1–5]. Individualised approach has been demonstrated as being particularly effective in implementing 3PM strategies in dentistry [6–8].

Furthermore, suboptimal health conditions and reversible damage are particularly attractive for the cost-effective targeted prevention in dentistry that is central for the healthcare at large [5, 6]. General chronic periodontitis (GCP) is a bacterial inflammatory disease that destroys the supporting structures of the teeth [9, 10] affecting the majority of adults worldwide and leading to associated systemic disorders in a reciprocal manner [11, 12]. Targeted prevention of GCP is an attractive focus of PPPM strategies in dentistry considering the tremendous socio-economic impact of the disorder [13].

### Periodontal disease as the prominent example requesting healthcare improvements

The fact of an evident underestimation of the targeted prevention of dental diseases is strongly supported by alarming epidemiologic statistics globally. For example, epidemiologists demonstrated 100% prevalence of dental caries in the Russian population followed by clinical manifestation of periodontal diseases [2]. In combating these trends, professional oral care and individualised medical services play an important role [14–17]. Furthermore, to raise the awareness in the area is essential to advance the overall efficacy of the oral care in the population [18, 19].

Inadequately provided oral health services in populations are caused by multi-factorial deficits including but not limited to the low socio-economic status of affected individuals (low educational level and income), lack of insurance in sub-populations, insufficient density of dedicated medical units [20, 21]. Another important aspect is the “participatory” medicine based on the active participation of population in maintaining oral health. To this end, healthcare will remain insufficient as long as the patient is not motivated and does not feel responsible for their oral health [17, 22–24]. Contextually, nearly half of chronically diseased people do not comply with adequate medical services suffering from severely progressing pathologies [25].

### Age as a potential determinant for patient stratification and tailored 3PM strategies

It is evident that individual age groups behave differently towards healthcare measures including the spectrum of medical services requested, their duration, quality and costs. Healthcare challenges in elderly should be discussed from various perspectives including medical, mental and social aspects; in particular, general “hypofunction” and “dysfunction” towards oral disorders have been reported for this sub-population [26–32]. Specific psychological features of this patient group should be taken into consideration such as losing physical and financial independence, amongst others [33, 34]. Adapted concepts of optimal oral care have been presented specifically for geriatrics and gerontology demonstrating that full recovery can be reached before irreversible frailty with symptoms of decreased articulation, choking/spillage at eating, and increasingly unchewed food. Still, this is considered to be a health condition, but not a disease. Contextually, specific measures of oral hygiene and dental prevention should be appropriately adapted to the needs of this age group preventing oral dryness, reduced occlusal force, decreased tongue-lip motor function, decreased tongue pressure, decreased masticatory function, and deterioration of swallowing function which collectively lead to the oral hypofunction with advanced ageing [40–42].

The aim of this article is to investigate the age-related aspects as a variable for adapted 3PM strategies, personalised programmes for prediction and prevention of diminishing oral health as well as co-prevention of related pathologies and poor individual outcomes.

## Working hypothesis

The current study hypothesised that age, gender, socio-economic status, quality of environment and life-style, oral hygiene quality, regularity of dental services requested, level of motivation and responsibility for own health status, and corresponding behavioural patterns are the key parameters for patient stratification considering person-tailored approach in a complex dental care in the population. If so, adapted screening programmes and treatment schemes are crucial for the complex person-tailored dental care to improve individual outcomes and healthcare provided to the population.

## Study design

### Patient recruitment and stratification

The study enrolled 706 dental patients (529 women and 177 men) aged between 18 and75 years. Potential gender-related differences have not been considered by current study. The recruited patients have been stratified by the age and according to the WHO recommendations [31] and have been grouped as follows: (A) between18 and 24, (B) between 25 and 44, (C) between 45 and 60, (D) between 61 and 75 years of age.

### Application of the specialised survey

The participants underwent a comprehensive survey developed at Therapeutic Dentistry and Periodontology Department, Pavlov First Saint Petersburg State Medical University specifically for the purposes of this study. The survey comprises the questions regarding.

1. The current status of the individual dental health
2. the lasting exposure to specific occupational hazards such as abrasives, acids, low and high temperatures, heavy physical activity, heavy metal salts
3. The individual attitude of the patient towards the oral cavity disease prevention and their general knowledge towards the most optimal oral care.

The specialised survey has been developed based on the expertise collected at Therapeutic Dentistry and Periodontology Department, Pavlov First Saint Petersburg State Medical University regarding the origin of the oral cavity diseases and corresponding protective measures which are in consensus with up-to-date international knowledge in the area as described in the literature [1, 3, 9, 14, 15, 31, 32, 34].

### Statistics

The descriptive statistics were performed presenting corresponding data as a percentage of total for each feature analysed. The groups were compared by the chi-square criterion. Multiple comparisons were analysed using the Holm-Bonferroni method. Statistical estimates were carried out using LBM SPSS Statistics 20.0. Statistically significant values were considered by *P* < 0.05.

## Results

### Recruited respondents reflect well the demographic profile of visitors to the dental care specialists

**Fig. 1:**
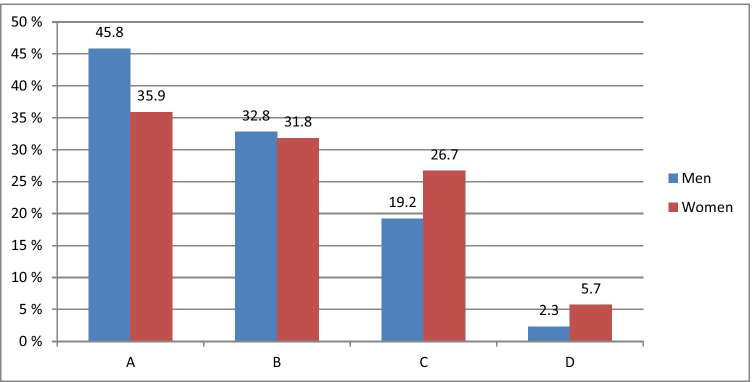
Respondents stratified by age and gender

Figure 1 demonstrates stratification of 706 patients by age and gender recruited for the study and underwent the specialised survey.

The stratified age groups differ significantly (*P* < 0.001) from each other regarding the frequency of their visits to the dental care specialists that reflects well general statistics monitored by dental care centres in St. Petersburg, Russia. To this end, the most frequent visitors were young people aged between 18 and 24 years (35.9% women and 45.8% men). In contrast, the rarest visitors were people aged between 61 and 75 years (5.7% women and 2.3% men). Regularity and reasons of dental visits by patients in different age groups are outlined in Table 1. One-third of the respondents within the participating groups visited a dentist twice a year. Patients aged between 61 and 75 years visited dentists less than once a year.

**Table 1:** Frequency and reasons of dental visits by patients stratified by age. *P*_AB_—level of significance of difference between groups A and B; *P*_AC_—level of significance of difference between groups A and C; *P*_AD_—level of significance of difference between groups A and D; P_BC_—level of significance of difference between groups B and C; P_BD_—level of significance of difference between groups B and D; *P*_CD_—level of significance of difference between groups C and D

No	Indicator		18 to 24 years old, *N* = 271	25 to 44 years old, *N* = 226	45 to 60 years old, *N* = 175	61 to 75 years old, *N* = 34	Significance *P*
	Group		A	B	C	D	
1	Frequency of dental visits	Less than once a year	34 (12.5%)	39 (17.3%)	46 (26.3%)	12 (35.3%)	*P*_AB_ > 0.05 *P*_AC_ < 0.01 *P*_AD_ < 0.01 *P*_BC_ > 0.05 *P*_BD_ > 0.05 *P*_CD_ > 0.05
		Less than twice a year	54 (19.9%)	34 (15.0%)	27 (15.4%)	3 (8.8%)	
		Once a year	84 (31.0%)	76 (33.6%)	53 (30.3%)	7 (20.6%)	
		twice a year	99 (36.5%)	77 (34.1%)	49 (28.0%)	12 (35.3%)	
2	Reason for dental visits	Routine oral cavity treatment	72 (26.6%)	92 (40.7%)	65 (37.1%)	13 (38.2%)	*P*_AB_ > 0.05 *P*_AC_ > 0.05 *P*_AD_ > 0.05 *P*_BC_ < 0.001 *P*_BD_ < 0.01 *P*_CD_ < 0.01
		Acute pain	24 (8.9%)	20 (8.8%)	35 (20.0%)	6 (17.6%)	
		Preventive check-up	175 (64.6%)	114 (50.4%)	75 (42.9%)	15 (44.1%)	

Table 1 shows that the routine oral cavity treatment is more often taken by patients between 25 and 44 years old (40.7%). Consequently, in this group, the number of visits to a dentist with acute pain is lower than in any other group of comparison. Young people (between 18 and 24 years old) are most frequent visitors for preventive check-up.

The largest portion of respondents who used to seek medical treatment of acute toothache was registered for groups between 45 and 60 (20%) and between 61 and 75 (17.6%) years old that corresponds well with the fact that only one-third of them take routine oral cavity treatments.

### Chronic disorders in stratified patient groups

**Fig. 2:**
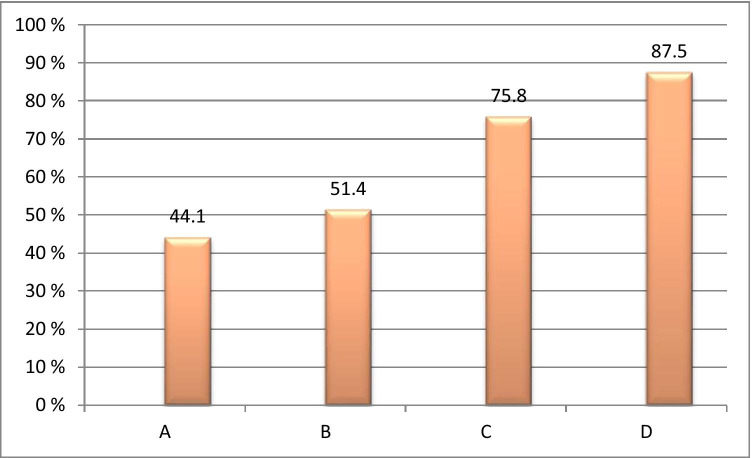
Frequency of chronic disorders in stratified patient groups; cardiovascular diseases, diabetes, gastrointestinal diseases, chronic kidney disease, and liver disease, amongst others, have been considered; significance of a difference between all groups involved is *P*_ABCD_ < 0.001

Responders have been analysed towards their collateral chronic disorders such as cardiovascular diseases, diabetes, gastrointestinal diseases, chronic kidney disease, and liver disease, amongst others. Figure 2 summarises collected data.

Respondents in the oldest group (between 61 and 75 years old) demonstrate the highest level of chronic diseases, namely 87.5% followed by the group between 45 and 60 years old (75.8%).

### Oral care preferences in stratified patient groups

**Fig. 3:**
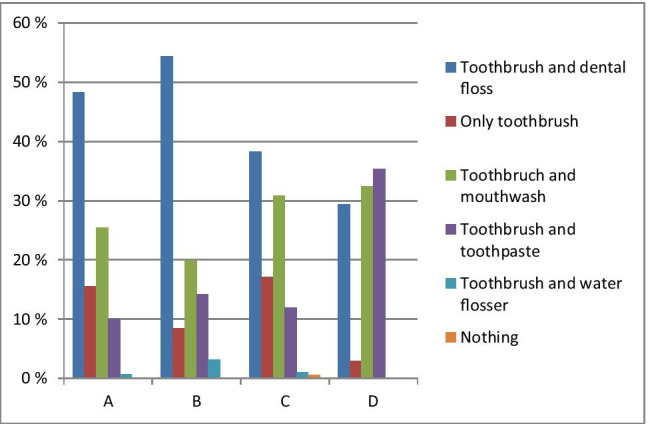
Oral care preferences in the stratified patient groups; significance of a difference between groups A and B is *P*_AB_ < 0.05; significance of a difference between groups A and C is *P*_AC_ > 0.05; significance of a difference between groups A and D is *P*_AD_ < 0.001; significance of a difference between groups B and C is *P*_BC_ < 0.01; significance of a difference between groups B and D is *P*_BD_ < 0.01; significance of a difference between groups C and D is *P*_CD_ < 0.05

Collected statistics demonstrate that 80.2% of respondents in all groups taken together, clean their teeth twice a day. However, a significant difference (*P* < 0.05) has been observed between the oldest and youngest groups of patients, namely 29.4% versus 11.1%, respectively. More than a half of respondents replace the toothbrush every 2 months. However, some of them replace the toothbrush only if it wears out or broken, that is more typical for the oldest group (38.1%). The stratified patient groups differ significantly in their preferences towards oral care approach and products as demonstrated in Fig. 3.

**Fig. 4:**
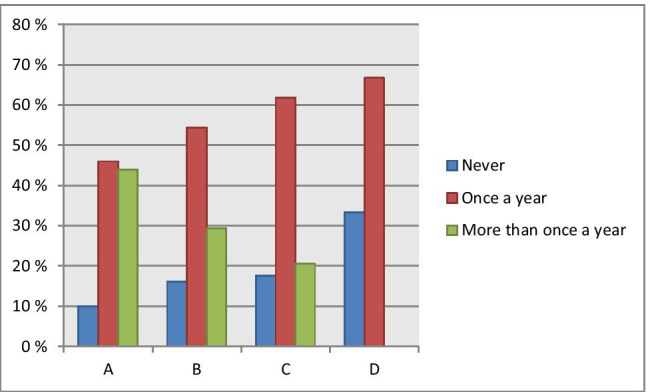
Regularity of professional oral care in stratified patient groups: Particular difference was observed considering the frequency of professional oral care used by youngest and oldest groups: either “never” or “once a year” was most characteristic for the oldest group compared to all other groups, whereas “more than once a year” was the most characteristic for the youngest group; significance of a difference between groups A and B is *P*_AB_ > 0.05; significance of a difference between groups A and C is *P*_AC_ > 0.05; significance of a difference between groups A and D is *P*_AD_ < 0.01; significance of a difference between groups A and D is *P*_BC_ > 0.05; significance of a difference between groups B and D is *P*_BD_ > 0.05; significance of a difference between groups C and D is *P*_CD_ > 0.05

Only a toothbrush and toothpaste are used by 10%, 14.2%, 12% and 35.3% of responders in groups A, B, C and D, respectively, and only toothbrush is used by 15.5%, 8.4%, 17.1% and 2.9% in corresponding groups. Floss is more frequently used by younger responders in contrast to the mouthwash preferred rather by the oldest group. Noteworthy, 0.6% of responders in group C do not make any use of the oral hygiene measures and instruments (Fig. 4).

## Data interpretation in the context of 3P medicine

### Periodontal and systemic diseases—the functional link

Periodontal diseases (PDs) are widespread in both developed and developing countries and affect about 20–50% of world populations. PDs are prevalent through the entire age spectrum from adolescence to elderly challenging therefore global public healthcare systems [35]. Periodontal disease is characterised by the destruction of connective tissues of the periodontium and alveolar bone that may lead not only to local symptoms, but also to systemic diseases and/or their complications, such as cardiovascular diseases, diabetes, liver disease, chronic obstructive pulmonary disease and several types of cancer [36]. Proposed mechanisms include bacteraemia and concomitant systemic inflammation, elevated C-reactive protein patterns and imbalanced oxidative stress [3, 36]. Risk factors such as poor oral hygiene, irregular dental care, diabetes mellitus, smoking, ageing, medication and stress overload—individually and synergistically—promote PD development in populations [35]. Furthermore, a robust evidence demonstrates a reciprocal relationship between PD development and systemic diseases including but not restricted to systemically altered microbiome and inflammation [3], chronic kidney disease [3, 36], neurodegenerative pathologies [37] as well as bacterial superinfections, pneumonia and sepsis characteristic for viral epidemics such as the actual COVID-19 pandemic condition (see the dedicated subchapter provided below). PDs significantly increase risks of cardiovascular diseases and mortality rates in patients with co-incidence of diabetic history and severe PD forms compared to no or mild PD [38]. Periodontitis is highly relevant for maternal infections, preterm birth, low birth weight and preeclampsia [35].

Although PDs are characterised by a local inflammatory process, several studies have shown that inflammatory mediators produced during this process, as well as subgingival species and bacterial components, can get disseminated from the oral cavity leading, therefore, to various extra-oral diseases including systemic inflammation and cancers [38]. To this end, carcinogenesis associated with periodontal species has been observed in both the oral cavity and in extra-oral sites known as the “oro-digestive” cancer types: oral, oesophageal, gastric, colonic and pancreatic malignancies [38].

### Association between periodontal diseases, bacterial overload and poor outcomes of viral infections: lessons for protective measures under pandemics

#### Experience collected in the past with influenza outbreaks

During influenza outbreaks, it has been observed that respiratory viruses were associated with bacterial superinfections as the common feature for a particularly severe disease course and the primary cause of death opposed to the virus itself as evident, for example, for influenza in 1918, H1N1 influenza in 2009 [39] and others. Periodontopathic microflora has been demonstrated as being implicated in imbalanced microbiome alterations, systemic inflammation and pneumonia development, in severe cases leading to sepsis and death. Contextually, the most optimal treatments utilise the dual antiviral and antibiotic medication [40]. This rich experience actually promotes extensive research activities to explore a potential association between the disease course severity and oral hygiene in COVID-19-infected individuals. Preliminary results have been reported during the year 2020.

#### Association between the disease course severity and oral hygiene in COVID-19-infected individuals

Clear association between diagnosed periodontitis and high risk of admission to intensive care units, need for assisted ventilation and increased COVID-19-related death have been demonstrated [41]. In consensus, the study performed in the UK has reported over 50% of deaths in COVID-19-infected patients exhibiting bacterial superinfections and the severe disease course [40]. High levels of *Prevotella*, *Staphylococcus* and *Fusobacterium* representing periodontopathic bacteria have been demonstrated specifically for the patient cohorts with poor COVID-19 outcomes. To this end, for 80% of patients treated at intensive care units, a particularly high oral bacterial load has been recorded.

Noteworthy, the prominent risk factors and comorbidities linked to the severe disease course and poor outcomes in COVID-19-infected individuals, such as elderly, diabetes mellitus, hypertension and cardiovascular disease, are frequently associated with significantly altered oral microbiome profiles, systemic inflammatory processes and poor oral health [42].

Suggested pathomechanisms consider potential preferences in the interaction between the viral particles and the host microbiota including oral cavity, the respiratory and gastrointestinal tracts [43]. Since an aspiration of periodontopathic bacteria induces the expression of angiotensin-converting enzyme 2—the receptor for SARS-CoV-2—and production of inflammatory cytokines in the lower respiratory tract, poor oral hygiene and periodontal disease have been proposed as leading to the COVID-19 aggravation [44].

Consequently, the issue-dedicated expert recommendations are focused on the optimal oral hygiene as being crucial for improved individual outcomes and reduced morbidity under the COVID-19 pandemic conditions [40, 45]. For an effective prevention, an application of oral probiotics has been proposed connecting the gut-lung axis with the viral and microbial pathogenesis, inflammation, secondary infections and severe complications linked to COVID-19 [46].

## Conclusions and expert recommendations in context of predictive, preventive and personalised (3P) medicine

### Conclusions

Current study demonstrated that age, gender, socio-economic status, quality of environment and life-style, oral hygiene quality, regularity of dental services requested, level of motivation and responsibility for own health status, and corresponding behavioural patterns are the key parameters for patient stratification considering the person-tailored approach in a complex dental care in populations. Consequently, adapted screening programmes and treatment schemes are crucial for the complex person-tailored dental care to improve individual outcomes and healthcare provided to the population.

###  Expert recommendations

#### Individualised patient profiling

Application of analytical instruments focused on phenotyping and genotyping of patients is highly recommended. For precise phenotyping, relevant questionnaires have been demonstrated to be of great clinical utility and cost-efficacy. One of the prominent examples in the area is the “dry mouth syndrome” phenotype relevant for xerostomia-associated complications and pathologies, the predisposition to which can be detected early in life in the reversible phase of health adverse effects followed by cost-effective targeted prevention [4]. Recently, the topic-dedicated study demonstrated significant predisposition to xerostomia in young groups of population demonstrating voice perturbations under stress conditions [5]. The needs of young populations in suboptimal health conditions are clearly in the framework of the 3PM concepts [47, 48].

Genotyping analytical tools to diagnose inflammatory processes in early stages are highly recommended for targeted treatments and cost-effective prevention in populations [49, 50].

Application of pre- and probiotics is considered to be effective to stabilise individual microbiome and to reverse negative effects of pathogenic microflora [51] that is particularly true for disorders associated with inflammatory processes [52, 53].

#### Innovative screening programmes

Common origin but individual outcomes is the major challenge for differential diagnostics, disease prediction and patient stratification based on individualised patient profiling [54]. Contextually, innovative screening programmes should get well equipped by novel approaches and high-tech tools such as liquid biopsy utilising biomarker patterns in body fluids and artificial intelligence (machine learning, disease modelling, etc.) to identify and stratify disease-predisposed individuals and to create an accurate person-tailored treatment regime [55–57].

To this end, bio-banking plays the key role in the era of big data, machine learning, disease prediction, targeted treatments and prevention [58].

#### Educational measures for professionals and stratified patient groups in populations

Educational measures play the key role in improving the medical care literacy, broad understanding and acceptance of innovative strategies of 3P medicine by professional groups and in the population [58]. It is expected that principles of participatory medicine as an essential element of 3PM and, consequently, an active role of patients in the treatment procedure may better motivate patients for taking responsibility for their health status and quality of individual outcomes [22].

#### Collaboration with policy-makers

Population health is in focus of predictive, preventive and personalised (3P) medical approach presented above. Consequently, collaboration with policy-makers leading to health policy advancement, development of innovative screening programmes triggered and financially supported by the state is pivotal for a significant progress in the area of dental care benefiting the healthcare system and society at large [59].

**Fig. 5:**
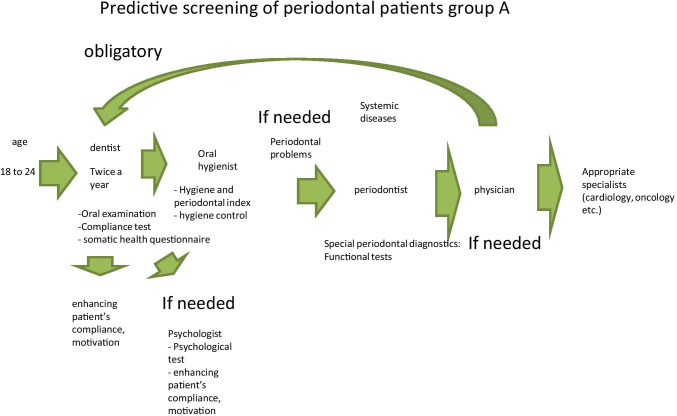
Predictive, preventive and personalised approach adapted to the specific needs of the stratified group A

**Fig. 6:**
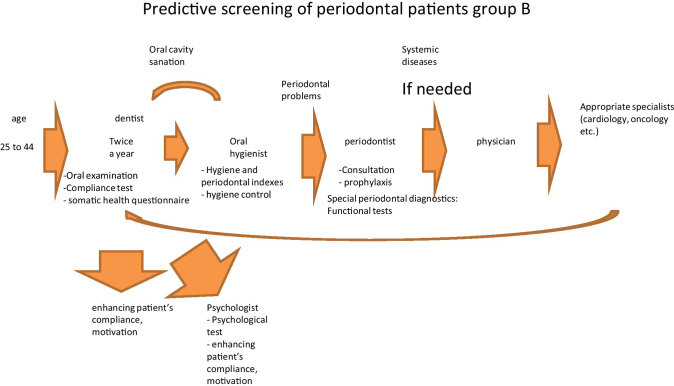
Predictive, preventive and personalised approach adapted to the specific needs of the stratified group B

**Fig. 7:**
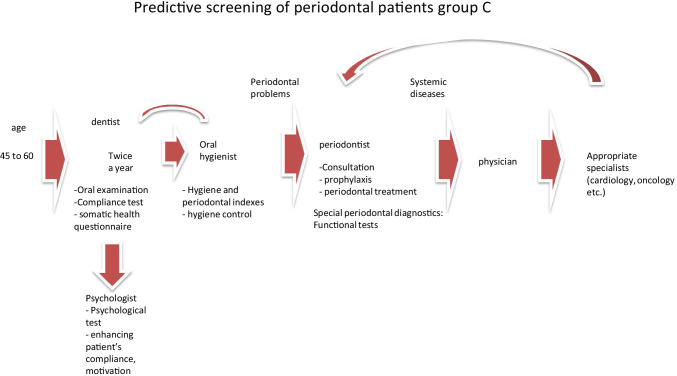
Predictive, preventive and personalised approach adapted to the specific needs of the stratified group C

**Fig. 8:**
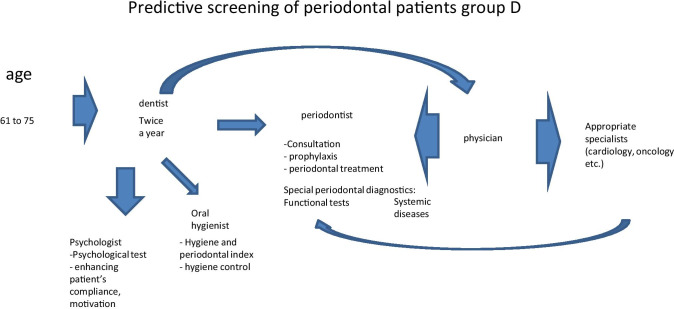
Predictive, preventive and personalised approach adapted to the specific needs of the stratified group D

Figures 5, 6, 7 and 8 present treatment schemes adapted to individual profiles and needs of stratified groups A, B, C and D.

The proposed treatment schemes differ from each other considering following group-specific aspects:

1. Group A patients have good oral and general health condition and compliance. Therefore, they mainly need to attend a dentist and dental hygienist for treatment, who in turn may re-address the patient to further specialists, if necessary;
2. Good general health condition, low compliance and rare periodontal problems are typical for group B patients; however, a regular periodontal consultation is necessary to monitor potential sub-optimal health conditions, wherefrom the patient may be re-addressed for the follow-up consultations by related medical experts, if necessary;
3. Group C patients usually have periodontal and general health problems, as well as low compliance. Therefore, they need in-depth physical examination and follow-up treatments tailored to the individualised patient profile;
4. Periodontal disease with frequent chronic collateral and related pathologies are the central problem of the group D. Therefore, periodontal and accompanied examinations followed by a comprehensive therapeutic approach are essential for this group presenting a severe economic burden.

## Declarations

All procedures performed in the study involving human participants were in accordance with the ethical standards of the institutional and/or national research committee and with the 1964 Helsinki declaration and its later amendments or comparable ethical standards. The study was approved by the Ethical Committee of the First Pavlov State Medical University of Saint Petersburg #01–2019. All patients were informed about the purposes of the study and have signed their “consent of the patient participation”.

## Abbreviations

PPPM/3PM: Predictive preventive personalised medicine
PDs: Periodontal diseases
WHO: World Health Organization
GCP: General chronic periodontitis
CVD: Cardiovascular disease
NCD: Non-communicable disease
